# Impact of Disease-Modifying Therapies on Gut–Brain Axis in Multiple Sclerosis

**DOI:** 10.3390/medicina60010006

**Published:** 2023-12-20

**Authors:** Ilaria Del Negro, Sara Pez, Salvatore Versace, Alessandro Marziali, Gian Luigi Gigli, Yan Tereshko, Mariarosaria Valente

**Affiliations:** 1 Clinical Neurology Unit, Udine University Hospital, Piazzale S. Maria della Misericordia, 33100 Udine, Italy; 2 Department of Medical Area (DAME), University of Udine, 33100 Udine, Italy

**Keywords:** multiple sclerosis, disease-modifying therapies, gut–brain axis

## Abstract

Multiple sclerosis is a chronic, autoimmune-mediated, demyelinating disease whose pathogenesis remains to be defined. In past years, in consideration of a constantly growing number of patients diagnosed with multiple sclerosis, the impacts of different environmental factors in the pathogenesis of the disease have been largely studied. Alterations in gut microbiome composition and intestinal barrier permeability have been suggested to play an essential role in the regulation of autoimmunity. Thus, increased efforts are being conducted to demonstrate the complex interplay between gut homeostasis and disease pathogenesis. Numerous results confirm that disease-modifying therapies (DMTs) used for the treatment of MS, in addition to their immunomodulatory effect, could exert an impact on the intestinal microbiota, contributing to the modulation of the immune response itself. However, to date, the direct influence of these treatments on the microbiota is still unclear. This review intends to underline the impact of DMTs on the complex system of the microbiota–gut–brain axis in patients with multiple sclerosis.

## 1. Introduction

Multiple sclerosis (MS) is a chronic, autoimmune-mediated, demyelinating, and degenerative disease that affects the central nervous system (CNS).

According to the International Multiple Sclerosis Federation, there are approximately 2.8 million people living with MS worldwide, with a trend of increasing prevalence since 2013 [1,2]. MS affects young adults between the ages of 20 and 40 and consequently imposes a large economic burden on society caused by lost productivity. Added to this are the costs for drugs, caregivers, and the healthcare system [3].

The exact mechanism underlying MS remains unclear, although inherited genetic components and environmental conditions play essential roles.

In recent years, a growing number of studies and amount of literature have focused on the relationship between intestinal microbial dysbiosis and the pathogenesis of MS [4,5].

For this reason, the analysis of the microbiota and its interaction with the immune system has largely been studied, and increasing interest has emerged in the complex mechanisms involved in the dysregulation of microbiota and intestinal barrier permeability.

MS treatment includes disease-modifying therapies (DMTs) that reduce the frequency of relapses and short-term disability. Recent studies with probiotics and short-chain fatty acids (SCFAs), produced mainly by some microbiota species, have shown possible synergistic effects with current MS therapy. These studies highlight how modifying the gut microbiota by dietary or medicinal approaches may represent an adjunctive therapeutic strategy for DMTs [6,7]. However, to date, the direct influence of DMTs on the microbiota is still unclear.

This article focuses on the interaction between microbiota, MS pathogenesis, and the mechanism of action of DMTs, summarizing the most compelling evidence regarding the effects and consequences of DMTs on the gut microbiome in MS patients.

## 2. Role of Intestinal Dysbiosis in the Pathogenesis of MS

Intestinal dysbiosis has been recognized as a constant feature during the clinical course of MS [8,9,10,11]. Microbiota changes have been associated with disease activity and an increase in pro-inflammatory T-helper 17 (Th17) immune responses or alterations in the gut-homing memory T cells correlated with developing the progressive phase of MS [12].

Multiple hypotheses speculate about the possible mechanisms involved in microbial dysbiosis and its impact on autoimmune disease: the enrichment or depletion of specific species of bacteria leading to intestinal inflammation, their interaction with the gut-associated lymphoid tissue (GALT), and increased intestinal permeability (“leaky gut”), which leads to the pro-inflammatory effect of metabolites of microbial origin on the CNS [4].

Researchers first discovered the association of brain autoimmunity with intestinal microbiota species in an experimental autoimmune encephalomyelitis (EAE) model. Mice kept in germ-free conditions or treated with oral broad-spectrum antibiotics developed a significantly less severe course of EAE than conventionally colonized mice [13,14].

Some species of commensal bacteria have been shown to play a dominant role in the contest of dysbiosis in MS. The bacterial composition in the human gut of healthy subjects and MS patients mainly belongs to the Firmicutes and Bacteroidetes phyla. The most significant number of the SCFA butyrate-producing bacteria belong to the Firmicutes phylum (notably *Faecalibacterium prausnitzii*); butyrate exerts an anti-inflammatory effect on epithelial cells, leading to a potent reinforcement of the intestinal barrier, thus preventing external or microbial antigens from systemic immune system presentation. In addition, Treg cell expression is inducted by butyrate [9]. Even if an increased total number of Firmicutes has been reported in MS patients, the anti-inflammatory SCFA-producing species (especially *Faecalibacterium* and *Clostridium*) are reduced in MS patients [9].

Bacteroidetes are Gram-negative anaerobic bacteria that exert a protective effect on microbiota functioning. Repeated evidence confirmed a reduction in these bacteria (with a marked impact on *Bacteroides* and *Prevotella*) in MS patients [8]. Bacteroidetes are the primary source of SCFA acetate and propionate acids, which are known to induce Treg functioning and subsequent reduction in Th1 and Th17 cells, thus leading to an anti-inflammatory effect on the immune system and an amelioration of autoimmunity [7,15]. Levels of lipid 654, an immunomodulatory ligand formed by certain Bacteroidetes species, also decreased significantly in the serum of MS patients compared to the control group, promoting autoreactive immune responses [16].

In the mice model, the polysaccharide A (PSA) of *Bacteroides fragilis* possesses a strong prophylactic effect in developing a severe EAE. PSA leads to the activation of dendritic cells (DC) of the local intestinal lymph nodes, stimulating the conversion of the cluster of differentiation (CD)4 T cells to FoxP3+ Treg cells, producing IL-10. Furthermore, in mice treated with purified PSA, decreases in CNS infiltration of Th1 and Th17 cells and in levels of interferon (IFN)-y and interleukin (IL)-17 were observed [17].

Conversely, filamentous bacteria of the family of Clostridiaceae, which promote IL-17-producing Th17 differentiation, were seen to be involved in developing pro-inflammatory T cells and a more severe EAE [18].

Another relevant factor to consider is the preservation of the intestinal barrier, which includes surface mucus, an epithelial layer, and immune defense mechanisms [19]. Translocation of lipopolysaccharide (LPS) and other metabolites, as well as whole bacteria, into the deep layers of the intestinal wall and local secondary lymphoid organs, which are the leading sites for the regulation of peripheral activated T cells and regulatory T cells, leads to the generation of circulating activated T cells. Moreover, alterations of the intestinal barrier are strongly related to an impaired blood–brain barrier (BBB) integrity, thus influencing the activation state of microglia and astrocytes [20,21,22,23].

Epsilon toxin (ETX) is a potent neurotoxic product of type B and type D *Clostridium perfringens*. This toxin can cross the intestinal barrier, reach and damage the endothelial cells of BBB, increase its permeability, and enter the brain. Numerous studies demonstrated the role of ETX in the direct damage to myelin and oligodendrocytes, with subsequent demyelination [24]. It has been hypothesized that MS patients may harbor type B and type D *Clostridium* species instead of type A (which typically colonizes the human gut and does not produce ETX), thus enhancing the toxin-mediated damage on CNS [25]. The altered integrity of these components leads to an increased intestinal permeability (so-called “leaky gut”).

Despite these findings, it has not yet been possible to accurately describe dysbiosis in MS, and the clinical relevance of gut microbiome alterations and their contribution to MS susceptibility remains controversial. The variability of the data may be partly explained by using different methods for stool collection or purification of microbial genes. For this reason, further large-scale studies of the MS microbiome with standardized technology are needed [26].

**Figure 1:**
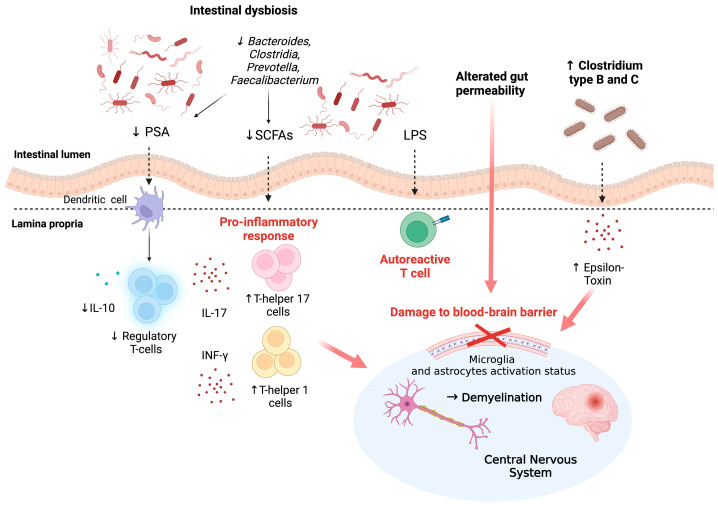
Role of intestinal dysbiosis in the pathogenesis of MS. SCFA-producing anti-inflammatory species (particularly *Faecalibacterium* and *Clostridia*) are reduced in MS patients, contributing to the downregulation of regulatory T cell expression and promotion of Th1 and Th17 cell activation. Impaired intestinal permeability in MS patients leads to an increase in autoreactive T cells and a reduced integrity of the BBB [10,11,13,17,18,19]. Translocation of bacterial metabolites and ETX directly affects resident CNS cells (especially microglia and astrocytes) with myelin damage and subsequent demyelination [24,25]. Moreover, BBB breakdown allows activated Th cells and autoreactive immune cells to reach the CNS and intensify central inflammation [4,8]. Legend: MS: multiple sclerosis, PSA: polysaccharide A, SCFAs: short-chain fatty acids, LPS: lipopolysaccharide, IL-10: interleukin-10, IL-17: interleukin-17, INF-y: interferon-y, ETX: epsilon toxin, CNS: central nervous system, BBB: blood–brain barrier. Created by BioRender.com.

Figure 1 summarizes the main mechanisms through which gut dysbiosis could influence the microbiota.

## 3. DMT Treatment Options for MS

Over the last 10 years, the introduction of various pharmacological therapies for MS radically changed the course of the disease by reducing relapse activity and the accumulation of disability [27].

Currently available DMTs are divided, according to their efficacy, into moderate-efficacy DMTs (interferon beta (IFN-β), glatiramer acetate (GA), dimethyl fumarate (DMF), and teriflunomide (TEF)) and high-efficacy DMTs (sphingosine 1 phosphate receptor (S1PR) modulators, natalizumab (NAT), anti-CD20 monoclonal antibodies, cladribine (CLB), and alemtuzumab (ALZ)) [28,29]. The therapeutic choice between the various DMTs depends on several elements, including the clinical characteristics of patients, radiological data, safety, adverse events, tolerability, and current prescription guidelines.

### 3.1. Mild- to Moderate-Efficacy DMTs

DMTs with mild to moderate efficacy are generally older compounds that were the first ones approved to prevent clinical relapses [30]. Their safety profile is excellent, although their capacity to control MS inflammatory activity is modest [29]. They are usually the first choice in a so-called “escalation treatment strategy” for comorbid patients or in a low-aggressiveness disease context [31].

Even if some have been used for decades, their interaction with the gut microbiome is still uncertain. It is well known that certain drugs may alter the intestinal barrier. Interestingly, new mechanisms of action can be unraveled by investigating their effect on the gut microbiome [32]. Moreover, some DMTs exhibit a protective role on the epithelial layer of the lamina propria due to their anti-inflammatory and antioxidant effects.

#### 3.1.1. Interferon-Beta

Recombinant human interferon beta-1b (IFN β-1b) was the first DMT approved for MS [33]. Currently, three IFN subtypes are available for clinical use: IFN β-1b, IFN β-1a, and pegylated IFN β-1a [34]. Interferons are cytokines that mediate pro- and anti-inflammatory responses to pathogenic stimuli, such as viral and bacterial stimuli in the intestine [32].

The intestinal immune system constantly interacts with the local microbiota through the epithelium, stimulating endogenous IFN production [35]. It seems to promote an anti-inflammatory response in the gut, and it is crucial to maintain immune system homeostasis [36] and reduce inflammation by suppressing IL-1b production and inflammasome activity [37]. In MS, the primary therapeutic effect of IFN β is thought to be the enhancement of the pro-regulatory role of T cells [30]. Mouse models support the idea that gut microbiota stimulates DC to produce IFN β, which augments the proliferation of Treg cells in the intestine and promotes their pro-regulatory effects [38].

Treatment with IFN β was associated with increases in the relative abundances of *Prevotella* and *Sutterella* and decreases in the relative abundances of the *Sarcina* genera, acting as a normalizer against a pro-inflammatory microbiota [8].

One study analyzed the differences in gut microbiota composition between patients with MS, untreated and treated with IFN β-1b, and healthy controls. There were significant differences in the proportion of probiotic species, particularly *Prevotella copri*, between controls and untreated patients. However, these differences disappeared when compared to treated patients with IFN β-1b. This could imply that the clinical effect of IFN β-1b may be in part mediated by normalization of the gut microbiota [39].

Serum and feces levels of propionate are reduced in MS compared to controls. In a large multi-center study, a significant increase in serum propionic acid was found in MS patients treated with IFN-β. Propionic acid is an SCFA that promotes Treg cell induction and activity and is associated with disease course improvement [7]. The authors propose an additional mechanism of action of the drug by upregulating the SCFAs’ monocarboxylate transporter-1 (MCT1; SLC16A1), which increases the intestinal absorption of microbially-derived propionate [40]

#### 3.1.2. Glatiramer Acetate

GA is a myelin-basic protein analog first approved for relapsing–remitting MS in 1996 [24,36]. It is administered subcutaneously and is thought to switch T cells toward a less inflammatory Th2 subtype to reduce the polarization of naïve T cells to Th1 and Th17 and enhance Treg activity [41].

GA modulates DCs, antigen-presenting cells (APCs) found in gut epithelia, skin, and lungs. DCs exposed to GA have an impaired capacity to secrete pro-inflammatory mediators that promote Th1 differentiation and can induce Th2 cell activity and increase anti-inflammatory IL-10 levels [42].

GA seems to exert a protective effect on the intestinal barrier [32]. In murine models of inflammatory bowel disease (IBD), GA administration ameliorates clinical manifestation and histologic colon damage by reducing levels of pro-inflammatory tumor necrosis factor α (TNFα) and INFγ cytokines and by promoting anti-inflammatory transforming growth factor β and IL-10 [43]. Moreover, two studies found that the therapeutic effect of GA in IBD models probably depends on anti-inflammatory T cell induction [44,45].

In rat models, the maximum penetration capacity of GA is in the ileum, an essential site for Peyer’s patches, and the colon. The correct preservation of the drug from the proteolytic activity of the proximal intestine, i.e., with appropriate delivery formulations, could be crucial to maximize its local biological activity in the lower intestine [46].

Administration of GA was also associated with increases in relative abundances of *Prevotella* and decreases in the *Sarcina* and *Sutterella* genera [8,47]. *Prevotella histicola*, which suppresses disease in the animal model of EAE, is increased in patients who receive GA [48]. In addition, GA and dimethyl fumarate (DMF) are associated with a significant decrease in the relative abundance of two members of the Clostridia families: *Lachnospiraceae* and *Veillonellaceae* [47]. Other differences in microbiota composition compared with untreated subjects described in the literature include *Bacteroidaceae*, *Faecalibacterium*, *Ruminococcus*, *Lactobacillaceae*, *Clostridium*, and other Clostridiales [49]. Interestingly, GA has also shown antibacterial properties against Gram-negative organisms such as *Pseudomonas aeruginosa* [50].

#### 3.1.3. Teriflunomide

TEF is an immunomodulatory oral drug approved as a DMT for MS [30]. TEF blocks pyrimidine synthesis, interrupting the proliferation cycle of T and B cells, exerting a cytostatic effect and limiting their involvement in the inflammatory processes of MS pathogenesis [51].

TEF, fingolimod (FTY), and DMF inhibit the in vitro growth of ETX-secreting *Clostridium perfringens* types B and D, acting as bacteriostatic agents [52]. This could represent a new, insufficiently explored mechanism of TEF’s action in preventing MS disease activity [24].

In addition, TEF increases CD39+ Treg cells in murine GALT, which are protective against EAE in mice [53].

#### 3.1.4. Dimethyl Fumarate

DMF was approved in 2014 in Europe as a first-line oral treatment for MS [24]. Pharmacological effects are related to activating the nuclear erythroid-2-related factor 2 (Nrf2) transcription pathway and inhibiting the transcription nuclear factor kappa B (NFkB). This ubiquitous pathway works as a cell defense system against potential injuries derived from inflammatory and oxidative stress, which are considered triggers in the pathogenesis of immune-mediated inflammatory diseases such as MS [54]. Moreover, fumarates are known for their antimicrobial properties, resulting in a generalized decrease in the relative abundance of many taxa [55].

In Lewis rats, DMF was able to mediate in the duodenum a reduction in the toll-like receptor-4 expression in DCs and a reduction in IFNγ mRNA expression in the lamina propria; in the ileum was seen a concomitant increase in the Foxp-3+ Treg population and an increase in the CD4+CD25+ Treg population in Peyer’s patches. These modifications lead to a less inflammatory phenotype of T cells in the GALT [56]. The anti-inflammatory, antioxidant, and antibacterial effects are believed to promote the maintenance of the integrity and function of the intestinal mucosa and to increase the abundance of bacteria producing SCFAs, which have a potent immunoregulatory role, such as *Gemella*, *Roseburia*, *Bacillus*, and especially *Bacteroides* [57]. Another study found that DMF decreases the abundance of many taxa of the gut microbiota, particularly the phyla Firmicutes and Fusobacteria and the order Clostridiales, and demonstrated an increase in Bacteroidetes [47].

A 12-week pilot trial of oral DMF in patients with MS was not associated with significant alterations in the gut microbiota composition. Still, there was a trend toward a near-normalization of the low presence of butyrate-producing *Faecalibacterium* in MS patients [58].

Different studies suggested that DMF, together with FTY and TEF, can inhibit the growth of the ETX-secreting *Clostridium perfringens* through its antimicrobial effect [52].

Recently, an association has been observed between a baseline gut microbiota composition (presence of *Akkermansia muciniphilia* and absence of *Prevotella copri*) and the development of lymphopenia during treatment. *Bacteroides dorei* was also overrepresented in lymphopenia patients [59]. Based on these findings, it has been hypothesized that baseline microbiota composition may be a critical mediator of DMF lymphopenia, suggesting a more complex microbiome–therapy interplay.

**Figure 2:**
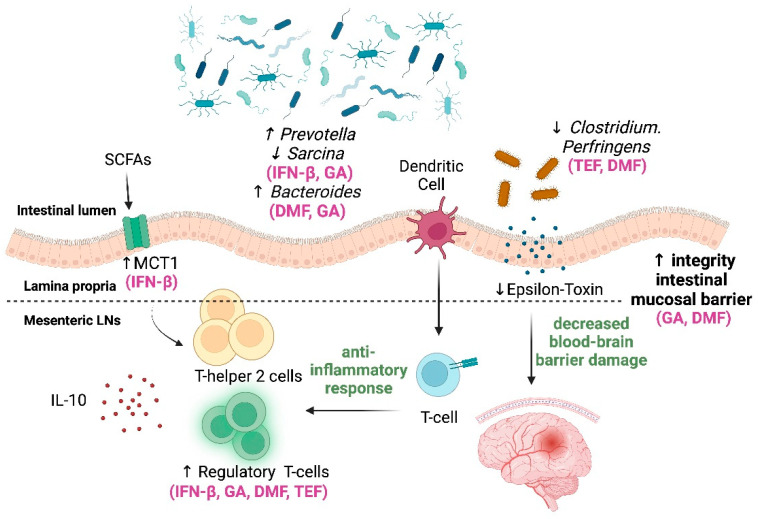
Impact of mild- to moderate-efficacy DMTs on gut–brain axis [35,36,39,42,43,44,45,46,47,50,51,52,53,56,57,58,60]. Legend: SCFAs: short-chain fatty acids, IL-10: interleukin-10, INF-β: interferon beta, GA: glatiramer acetate, DMF: dimethyl fumarate, TEF: teriflunomide. Created by BioRender.com.

The primary mechanism of action of mild- to moderate-efficacy DMTs is summarized in Figure 2.

### 3.2. High Efficacy DMTs

Although an objective definition of “high-efficacy disease-modifying therapy” (HEDMT) is still missing, many studies confirm that the early use of HEDMT represents a rewarding therapeutic strategy and is associated with a reduction in inflammatory activity and disease progression [60]. Since most of these drugs have been available only in recent years, knowledge about their interactions with the microbiome is lacking, and further studies are necessary.

#### 3.2.1. Sphingosine-1-Phosphate Receptor Modulators

S1PRs are high-affinity G-protein-coupled cell surface receptors. They have five distinct subtypes and are expressed throughout the body, mediating a broad range of biological functions, most of them implicated in immune cell trafficking and producing immune mediators [61]. S1PR modulators are oral DMTs approved as moderate- to high-efficacy therapies in MS. FTY was the first S1PR modulator approved for relapsing–remitting MS, followed by ozanimod and ponesimod. These drugs showed a reduced relapsing rate compared to placebos or moderate-efficacy DMTs [62,63,64]. Siponimod is the only S1PR modulator approved for secondary progressive MS [65]. These drugs act as functional antagonists or agonists of S1PR.

S1PR modulators have several effects on the gut inflammatory environment and have shown beneficial effects in the regulation and induction of CD4+CD25+ Treg cell activity [66] by protecting vascular integrity and blocking leucocyte migration through inflammatory mucosa [67]. Mouse models showed that the S1P-S1PR pathway mediates the migration of intraepithelial T lymphocytes (IELs). FTY can inhibit the trafficking of IELs into the intestinal epithelium and their retention in the mucosa [68].

In studies involving mice, S1PR modulators accumulated immunoglobulin (Ig)A+ plasma blast in Peyer’s patches, leading to their reduction in the lamina propria and impairing antigen-specific intestinal IgA production [69]. Intestinal IgAs play a critical role in the defense against external pathogens and in maintaining intestinal homeostasis, as they are part of the complex system necessary to maintain the correct gut permeability [70]. However, an exploratory study showed no reduction in secretory IgA in fecal samples of people with MS after long-term treatment with FTY [71].

In mice with type 1 diabetes, early FTY treatment was associated with good intestinal homeostasis maintenance and enhanced gut barrier integrity due to suppressing the local CD4+ cell population and the differentiation of Th1 cells [72].

In addition, in vitro studies have shown that FTY (as previously described for TEF and DMT) can inhibit the growth of the pathogen ETX of *Clostridium perfringens* [52].

#### 3.2.2. Natalizumab

NAT is an intravenous recombinant humanized IgG4 monoclonal antibody selective for α4-integrins, indicated for the highly active relapsing–remitting form of MS [73]. By preventing the interaction of α4β1 integrin (VLA-4) expressed on lymphocytes with its ligand vascular cell adhesion molecule 1 (VCAM1) on endothelial cells, the antibody inhibits both the migration through the BBB into the CNS parenchyma and the circulation of T cells in the gut [74].

Integrins have a leading role in leukocyte trafficking to the gastrointestinal tract. Targeting α4β1 integrin with NAT and integrin α4β7 (which plays an essential role in immune surveillance of the gastrointestinal tract by enabling lymphocytes to travel across the vascular endothelial barrier to the GALT or intestinal lamina propria) with NAT or vedolizumab are efficient therapeutic approaches in IBDs [75]. The inhibition of T cell trafficking in the gut exerts a protective anti-inflammatory effect and reduces the exposure to antigens of microbial origin; how these modifications could modify the gut microbiota has yet to be defined.

A study of 26 MS patients and 39 healthy controls showed that the altered gut microbiome of MS patients (with a decreased abundance of *Coprococcus*, *Clostridium*, or an unidentified *Ruminococcaceae* genus) led to an increased expression of C-X-C motif chemokine receptor 3 (CXCR3) in both CD4+ and CD8+ T cells, an increased expression of the gut-homing α4β7 integrin receptor [76], and more pronounced intestinal T cell trafficking.

Interactions between α4 integrins, the microbiome, and autoimmune diseases have also been studied in the diabetes type 1 murine model: mice with α4 integrin deletion did not develop the autoimmune disease, and α4− mice bone marrow transplantation in diabetic α4+ mice prevented diabetes relapses. However, α4+ mice showed abnormal microbiota after the first relapse, but since the α4− mice microbiota was indistinguishable from pre-disease α4+ mice, α4 could not modulate the microbiota, and the dysbiosis could be a consequence of the disease [77].

Studies on Rhesus monkeys show that antibiotic treatment resulting in the absence of commensal bacteria led to an upregulation of immune communication genes, including VCAM1. The innate immunity-associated genes (including VCAM1) demonstrated cross-talk and upregulation in response to bacterial dysbiosis, activating the major histocompatibility complex (MHC) system and T cell activity [78].

#### 3.2.3. Anti-CD20 Monoclonal Antibodies

Three anti-CD20 monoclonal antibodies are available for MS: ocrelizumab, ofatumumab, and the off-label use of rituximab [79,80,81]. CD-20 B cells are a main target of MS therapies due to their prevalence in the cerebrospinal fluid and the brain lesions of MS patients and their possible association with intrathecal inflammation and Ig synthesis. New evidence suggests that B cells have an antibody-independent function: they act as APCs to T cells, promoting T cell activation and proliferation. They also interact with APCs, influencing antigen trafficking by modulating anti- and pro-inflammatory cytokines and chemokines [79].

B cells are implicated in IgA production in the gut mucosa; as described for S1PR, these antibodies have a crucial role in maintaining intestinal barrier homeostasis. IgA+-producing cells are dramatically reduced in the gut during EAE; likewise, IgA-bound fecal bacteria is reduced in MS patients during disease relapse [82]. IgA+ plasmablasts and plasma cells were identified in the lamina propria of patients during peripheral B cell depletion following rituximab, implying that B cells resident in the mucosa are not deleted by this treatment [83].

An observational longitudinal study of 24 patients with MS who were treated with ocrelizumab showed a pro-inflammatory dysbiosis before treatment; the treated patients showed a decrease in *Bacteroidales* (particularly concerning the abundance of *Parabacteroides*, *Bacteroides*, and *Prevotella*) and several members of pro-inflammatory bacteria such as *Proteobacteria* (including *Escherichia* and *Shigella*). This study confirmed the beneficial long-term effect of anti-CD20 depletion therapy on microbiota composition [84].

Studies in vitro and in vivo involving mice showed that integrating *Lactobacillus reuteri* alleviates rituximab’s gastrointestinal toxicity, reducing pro-inflammatory cytokines and increasing intestinal tight junction protein levels [85].

#### 3.2.4. Cladribine and Alemtuzumab

CLB and ALZ are immune therapies approved for MS that exert a transient immunodepletion followed by reconstitution and prolonged immunomodulation.

CLB is an oral drug with a pulsatile schedule. The active metabolite of CLB, 2-chlorodeoxyadenosine triphosphate, accumulates in the cells, disrupting cellular metabolism and inhibiting DNA synthesis and repair, with subsequent apoptosis. CLBs preferentially affect lymphocytes due to their relatively high ratio of deoxycytidine kinase to 5′-nucleotidase and to the fact that they are dependent on adenosine deaminase activity to maintain the equilibrium of cellular concentrations of triphosphorylated nucleotides [86]. The accumulation of the CLB metabolite produces rapid and sustained reductions in CD4+ and CD8+ cells and rapid, though more transient, effects on CD19+ B cells, with the relative sparing of other immune cells [87].

ALZ is an intravenous humanized anti-CD52 monoclonal antibody used for the treatment of MS that causes a depletion and subsequent repopulation of circulating T and B lymphocytes [88].

Both these drugs lead to changes in the number, proportion, and functions of some lymphocyte subsets [89], with a sensitive reduction in disease activity in relapsing–remitting MS and a long-term reassessment of the immune system [90,91].

In cynomolgus monkey models treated with ALZ, the intestinal microbiota composition was altered after the depletion of mucosal lymphocytes and the apoptosis of intestinal barrier endothelial cells. Sequenced samples following ALZ administration showed that some specific bacteria, from the orders Lactobacillales, Enterobacteriales (in particular *Escherichia coli* and *Shigella flexneri*), and Clostridiales were overexpressed; on the other hand, *Bacteroides* genus and *Faecalibacterium prausnitzii* were less abundant. These species were primarily susceptible to a transient alteration of the gut microbiota after lymphocyte depletion, causing dysbiosis, which, however, goes back to the previous state 35 days after the treatment [92]. In another study on monkeys, it was also shown that there was an increased diversity and colonization of fungal populations such as *Candida albicans*, *Aspergillus clavatus*, *Saccharomyces cerevisiae*, and *Botryotinia fuckeliana*. The fungal microbiota of colonic mucosa was restored concomitantly with T-lymphocyte reconstitution [93].

A prospective study, whose goal is to examine if the changes in gut and oral microbiota and associated changes in the immune response are predictors for the response to treatment in subjects with active relapsing–remitting MS treated with oral CLB, will end in 2024 and will give us further information [94].

Despite the overwhelming effect of CLB and ALZ on the immune system, these drugs can lead to transient dysbiosis during lymphocyte depletion, the effect of which on gut immune tolerance has yet to be elucidated. Even if the impact of intestinal T cells on the microbiota remains quite undetermined, these studies confirm the substantial role of mucosal T-lymphocytes in maintaining microbial homeostasis.

**Figure 3:**
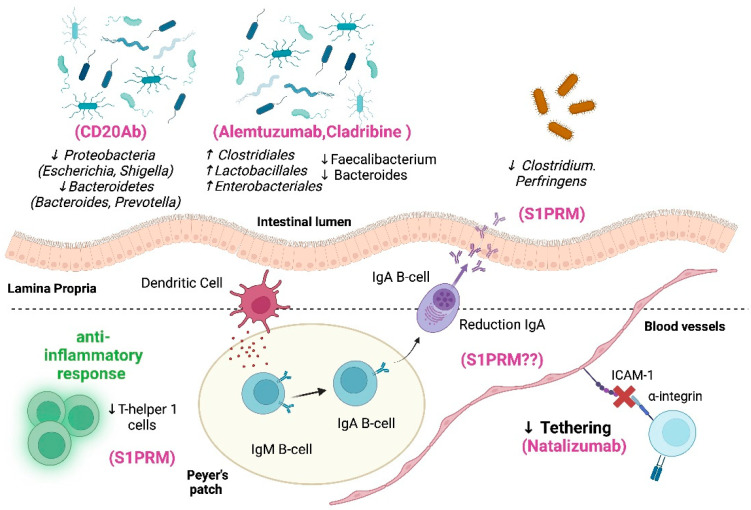
Impact of high-efficacy DMTs on gut–brain axis [52,66,67,68,69,71,72,75,76,83,92,93,94]. Legend: CD20Ab: anti-CD20 antibodies, S1PRM: sphingosine-1-phosphate receptor modulators. Created by BioRender.com.

Figure 3 graphically summarizes the main mechanism of action of high-efficacy DMTs on the gut–brain axis.

## 4. Discussion

In this literature review, we tried to clarify the complex interactions between DMTs and gut microbiota to better understand how they can influence each other.

MS is a chronic disease that affects millions of patients worldwide. Both genetic and environmental factors are thought to be involved in the development and progression of the disease. A better understanding of these mechanisms could lead to the refinement of current therapeutic approaches.

The natural history of MS has changed since the approval of DMTs, especially in recent years with the introduction of high-efficacy DMTs [27,28,29]. In parallel, there has also been an increasing interest in non-pharmacological interventions targeting the gut microbiota [9]. Emerging evidence shows that gut dysbiosis is involved in the development of numerous autoimmune diseases, including MS. A key role is played by SCFA-producing strains, which tend to be reduced in MS patients, losing their anti-inflammatory effect and their preserving role of the intestinal epithelium. Moreover, many different bacterial metabolites can pass through a “leaky” gut barrier, activating the local and systemic immune response and reaching the CNS where they can induce the demyelinating processes [8,9,10,11]. In this context, promoting a state of eubiosis could hypothetically play a role in preventing the onset of MS, treating its relapses, and slowing its progression. Nevertheless, the extreme variability of human intestinal microbiota and the participation of various bacteria in different metabolic pathways add much complexity to the definition of “healthy” microbiota. The impact of DMTs on gut microbiota in MS patients has been largely investigated. Studies are limited due to the small number of participants and the heterogeneity of the series and samples. However, although with different mechanisms, several results confirm that DMTs, in addition to their immunomodulatory effect, exert an impact on gut microbiota, which can, on the other hand, be part of regulating the immune response itself.

Most DMTs, particularly for mild- to moderate-efficacy therapies, can positively affect microbiota and partially or entirely reverse the chronic dysbiosis observed in MS patients. It is emblematic that IFN β, the first DMT to be approved for MS, is analogous of the endogenous IFN, which increases the activity of regulatory T cells and promotes an anti-inflammatory environment in the gut [35]. This suggests that the effect of IFN-β could also be mediated by its anti-inflammatory influence on gut microbiota.

GA appears to have an important role in modulating the intestinal flora, switching T cells to a less inflammatory subtype, protecting the intestinal barrier, and reducing Clostridia families [42,43,44,45,50], while clinical studies on TEF are very limited [53].

DMF emerges as the DMT with the broadest spectrum of positive effects on the gut–brain axis, acting on gut barrier integrity and favoring SCFA-producing strains [56,57,58]. On the other hand, highly effective DMTs exert a more substantial impact on disease activity and inflammation, but evidence regarding their role on gut dysbiosis is still limited and controversial, partly due to their recent availability in clinical practice. Among high-efficacy DMTs, anti-CD 20 monoclonal antibodies and S1PRs appear to have a greater potential to exert an anti-inflammatory action at the level of intestinal flora [83,84,85]. On the other hand, both CLB and ALZ can produce transient dysbiosis during the administration time and the phase of lymphocyte depletion [92,93,94].

Although these results are very intriguing, it is still unclear if the gut microbiota changes after the DMT administration should be considered only spectators of DMT action or, on the contrary, if they can help and increase DMT efficacy.

The main effects of all the DMTs discussed in this review are summarized in Table 1 and Table 2.

## 5. Conclusions

Numerous results confirm that DMTs used for the treatment of MS have a significant impact on the gut–brain axis.

Since the intestinal microbiota affects both local and systemic immune responses, the changes that DMTs induce in commensal microbiota composition could be associated with different levels of treatment effectiveness and side effects among patients. Furthermore, in choosing the optimal immunomodulatory treatment, the gut microbiota of the individual patient could represent a biomarker predicting a positive response to a specific DMT. Confirmation of these hypotheses by future studies could play an essential role for tailored medicine.

## 6. Future Directions

Current studies are based on small sample sizes and future randomized controlled trials will have to evaluate a larger population to better understand the effect of DMTs on the composition of intestinal microbiota. Furthermore, most of the research studies analyzed in our review involve experimental animal models and were not conducted on human diseases. The adoption of standardized protocols for the collection of fecal samples in humans is a possible strategy that facilitates more reliable results.

In addition to this, we noticed that the majority of the studies lack comparisons between single DMTs and long-time longitudinal follow-ups. Long-term evaluation would allow us to consider patients with similar clinical courses in order to better evaluate the role of the intestinal microbiota in the pathophysiology of relapse and in the efficacy of DMTs.

Extensive knowledge in the field could lead to the introduction of gut-microbiota-restoring therapies, such as SCFAs, probiotics, or dietary guidelines, into the clinical practice of MS patients.

## Figures and Tables

**Table 1:** Principal mechanism of action on gut–brain axis of different DMTs.

DMT	Main Effects on Gut–Brain Axis
Interferon beta [8,38,39,40]	Direct enhancement of pro-regulatory T cells
	Increased level of SCFAs
Glatiramer acetate [41,42,43,44,45,46,47,48,49,50]	Switch of T cells to a less inflammatory subtype
	Protective role on the intestinal barrier
	Reduction in Clostridia families
Teriflunomide [52,53]	Inhibition of ε toxin-secreting *C. perfringens*
	Increase Treg in GALT
Dimethyl fumarate [52,56,57,58,59]	Improvement of integrity of intestinal barrier (Nrf2, NFkB)
	Increase in SCFA-productor species (*Bacteroides*)
	Decrease in *Clostridium* species
	Increase in Treg in Payer’s patches and GALT
Sphingosine-1-phosphate receptor modulators [52,66,67,68,69,70,71,72]	Induction of Treg activity in the gut mucosa
	Inhibition of epsilon toxin-secreting *Clostridium perfringens*
Natalizumab [75,76,77]	Reduction in T-cell trafficking in the gut mucosa
Anti-CD20 monoclonal antibodies [82,83,84,85]	Maintenance of intestinal barrier homeostasis
	Increase in anti-inflammatory species and decrease in inflammatory species
Cladribine/Alemtuzumab [92,93,94]	Alteration of microbiota following mucosal lymphocyte depletion
	Alteration of intestinal barrier permeability

Legend: DMT: disease-modifying therapy, SCFAs: short-chain fatty acids, Treg: regulatory T cells, GALT: gut-associated lymphoid tissue.

**Table 2:** Schematic summary of typical effects on microbiota between different DMTs.

DMT	Increase in SCFAs-Producing Species	Decrease in Inflammatory Species	Reduction in Intestinal Permeability	Peripheral/GALT Treg Cells Regulatory Effect	Anti-Inflammatory T Cell (Lower Th1 and Th17, Higher Th2)	BBB and Oligodendrocytes Damage (ETX)
IFN [8,38,39,40]	++	+		++		
GA [41,42,43,44,45,46,47,48,49,50]	++	++	++		++	
TEF [52,53]				++		++
DMF [52,56,57,58,59]	++	++	+	++	++	++
S1PRM [52,66,67,68,69,70,71,72]			++	++	++	++
NAT [75,76,77]			++			
Anti-CD20 Ab [82,83,84,85]	#	++	+			
CLB/ALZ [92,93,94]	#	#	+	+		

# Opposite effect. Legend: DMT: disease-modifying therapy, SCFAs: short-chain fatty acids, GALT: gut-associated lymphoid tissue, Treg: regulatory T cells, Th1: T-helper-1, Th17: T-helper-17, Th2: T-helper2, ETX: epsilon toxin, IFN: interferon, GA: glatiramer acetate, TEF: teriflunomide, DMF: *Dimethyl fumarate*, S1PRM: Sphingosine-1-phosphate receptor modulators, NAT: natalizumab, anti-CD20 Ab: anti-CD20 antibodies.

## Data Availability

No new data were created or analyzed in this review.
